# Effects of lipid metabolism on mouse incisor dentinogenesis

**DOI:** 10.1038/s41598-020-61978-0

**Published:** 2020-03-20

**Authors:** Yutaro Kurotaki, Nobuhiro Sakai, Takuro Miyazaki, Masahiro Hosonuma, Yurie Sato, Akiko Karakawa, Masahiro Chatani, Mie Myers, Tetsuo Suzawa, Takako Negishi-Koga, Ryutaro Kamijo, Akira Miyazaki, Yasubumi Maruoka, Masamichi Takami

**Affiliations:** 1 Division of Community-Based Comprehensive Dentistry, Department of Special Needs Dentistry, School of Dentistry, Showa University, 2–1–1 Kitasenzoku, Ota, Tokyo 145–8515 Japan; 2 Department of Pharmacology, School of Dentistry, Showa University, 1–5–8 Hatanodai, Shinagawa, Tokyo 142–8555 Japan; 3 Pharmacological Research Center, Showa University, 1–5–8 Hatanodai, Shinagawa, Tokyo 142–8555 Japan; 4 Department of Biochemistry, School of Medicine, Showa University, 1–5–8 Hatanodai, Shinagawa, Tokyo 142–8555 Japan; 5 Division of Rheumatology, Department of Medicine, School of Medicine, Showa University, 1–5–8 Hatanodai, Shinagawa, Tokyo 142–8555 Japan; 6 Division of Dentistry for Persons with Disabilities, School of Dentistry, Showa University, 2–1–1 Kitasenzoku, Ota Tokyo, 145–8515 Japan; 7 Department of Biochemistry, School of Dentistry, Showa University, 1–5–8 Hatanodai, Shinagawa, Tokyo 142–8555 Japan; 8 Division of Mucosal Barriology, International Research and Development Center for Mucosal vaccines, The Institute of Medical Science, The Institute of Medical Science The University of Tokyo, 4-6-1 Shirokanedai, Minato, Tokyo 108-8639 Japan

**Keywords:** Lipoproteins, Dental pulp

## Abstract

Tooth formation can be affected by various factors, such as oral disease, drug administration, and systemic illness, as well as internal conditions including dentin formation. Dyslipidemia is an important lifestyle disease, though the relationship of aberrant lipid metabolism with tooth formation has not been clarified. This study was performed to examine the effects of dyslipidemia on tooth formation and tooth development. Dyslipidemia was induced in mice by giving a high-fat diet (HFD) for 12 weeks. Additionally, LDL receptor-deficient (*Ldlr*^*−/−*^) strain mice were used to analyze the effects of dyslipidemia and lipid metabolism in greater detail. In the HFD-fed mice, incisor elongation was decreased and pulp was significantly narrowed, while histological findings revealed disappearance of predentin. In *Ldlr*^*−/−*^ mice fed regular chow, incisor elongation showed a decreasing trend and pulp a narrowing trend, while predentin changes were unclear. Serum lipid levels were increased in the HFD-fed wild-type (WT) mice, while *Ldlr*^*−/−*^ mice given the HFD showed the greatest increase. These results show important effects of lipid metabolism, especially via the LDL receptor, on tooth homeostasis maintenance. In addition, they suggest a different mechanism for WT and *Ldlr*^*−/−*^ mice, though the LDL receptor pathway may not be the only factor involved.

## Introduction

A regular high-fat diet (HFD) has been shown to result in such lifestyle diseases as dyslipidemia, obesity, and diabetes^1,2^. Notably, dyslipidemia causes serious alterations of systemic tissue, including accumulation of lipids in blood vessel walls and the liver^3,4^. It has also been reported that lipid metabolism alteration produces changes in calcified tissue phenotypes^5^. Low-density lipoprotein (LDL) is known to transport cholesterol produced in the liver to peripheral cells^6^. However, as noted above, an increase in native LDL in blood induces dyslipidemia and atherosclerosis, which then induces typical cardiovascular events such as cardiac and cerebral infarction^7^. Increasing evidence shows that lifestyle, especially diet, can induce chronic disease. Furthermore, a previous study showed that patients with dyslipidemia can develop osteoporosis^8–10^. The gene disorder related to lipid metabolism known as familial hypercholesterolemia (FH) has been shown to be related to development of coronary heart disease as well as the LDL receptor, which is essential for lipid uptake^11,12^. Also, patients with FH have been reported to develop Achilles tendon (AT) xanthomas, resulting in an incrassate AT condition^13,14^, suggesting increased collagen in the tendon. However, detailed analysis findings regarding bone or teeth in FH patients are scarce. There are some reports of the relationship of lipids and teeth based on analyses of the components of enamel^15–17^ and dentin^17–20^, though details regarding the effects of lipid metabolism aberration on tooth formation and homeostasis maintenance remains unclear. Nevertheless, various factors, such as oral disease, drug administration, and systemic illness, are known to have effects on dentin and enamel formation.

Tooth aberrations, including hypodontia^21^, macrodontia^22^, microdontia^21^, and dentin dysplasia^20,23^, as well as others, mainly occur due to genetic and environmental factors, though few studies have investigated the latter, such as nourishment and systemic illness. Teeth are a type of highly calcified tissue that exist within an organism and composed of three forms of calcified hard tissue (enamel, dentin, cementum), while they also possess connective tissue known as pulp with abundant blood vessels and nerves. Dental pulp has important functions, including dentin formation, establishment of blood flow, tooth nourishment, and pain signaling^24,25^, and has been shown to have properties similar to mesenchymal stem cells^26,27^. In the outermost portion, odontoblasts exist and form a layer, then continuously produce dentin in pulp throughout life^24^. Initially, odontoblasts form predentin, an uncalcified tissue, after which predentin functions to mediate between dentin and odontoblasts.

A variety of good mouse models are available for analysis of incisor tooth development and aberrations. Previous results have shown that in the cervical loop region, stem cells are fueled as the incisor elongates continuously throughout the life of a mouse^28,29^. LDL receptor-deficient (*Ldlr*^*−/−*^) mice are generally used as animal models of dyslipidemia, atherosclerosis, and FH^30,31^. Dyslipidemia is induced in these animals by feeding an HFD and findings obtained with a dyslipidemia model indicated increases in total cholesterol (T-CHO), low density lipoprotein-cholesterol (LDL-c), and triglycerides (TG), as well as decreased HDL as compared to *Ldlr*^*−/−*^ mice fed normal chow^31^. In addition, the aorta of *Ldlr*^*−/−*^ mice fed an HFD narrows with accumulation of plaque. However, the influence of dyslipidemia on tooth growth has been scarcely reported. With this background in mind, we examined the effects of an HFD on tooth development and odontoblast activity. The aim of this study was to clarify the effects of dyslipidemia in mice induced by HFD consumption on tooth growth, histological alteration, tooth homeostasis maintenance, and bone metabolism.

## Results

### HFD-fed wild-type mice showed less incisor elongation and significantly narrowed incisor pulp as compared to chow-fed mice

**Figure 1:**
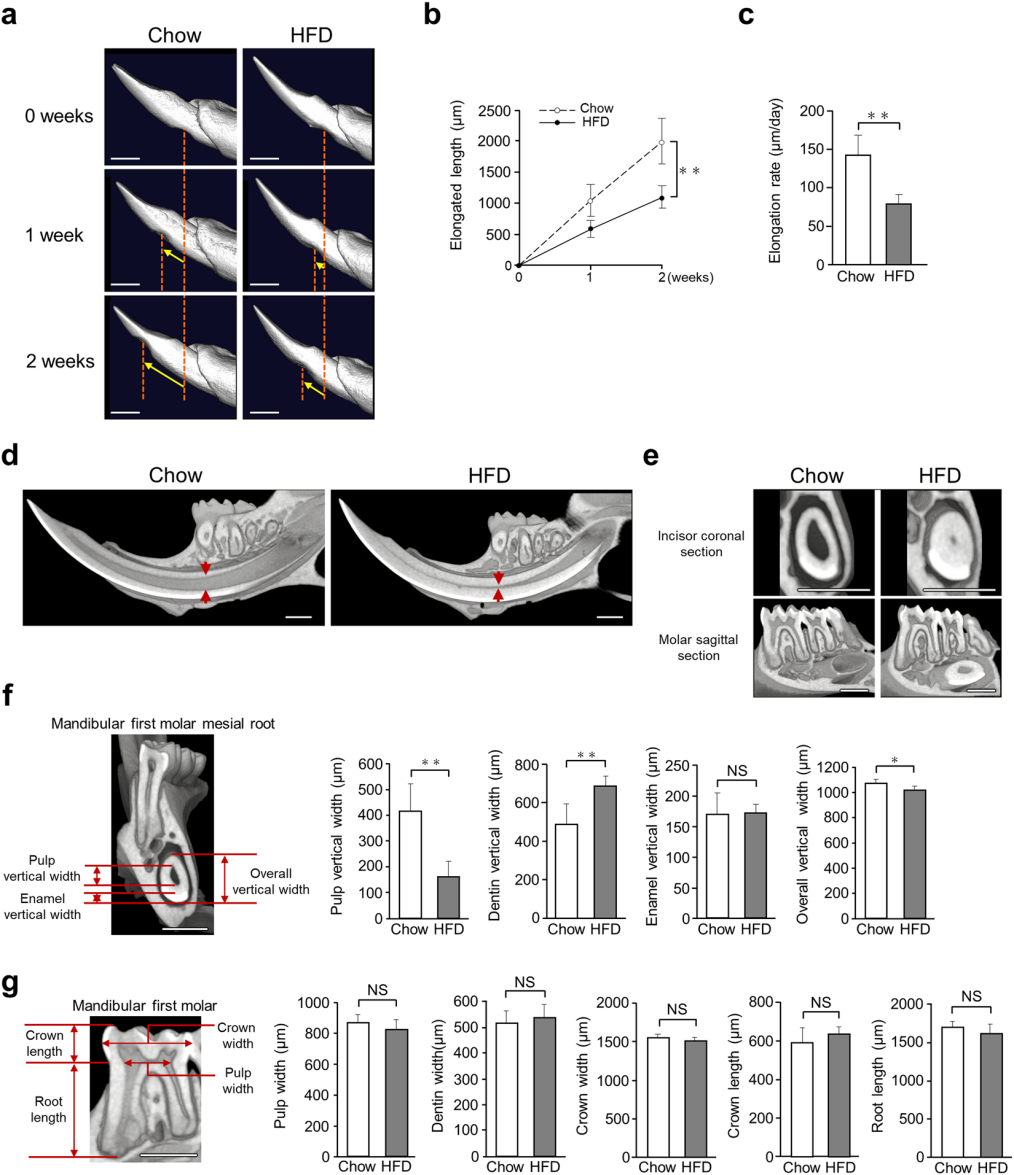
Determination of incisor elongation in chow- and high-fat diet (HFD)-fed wild-type (WT) mice, and micro-computed tomography (μCT) analysis of incisor inner structure. (**a**) μCT findings revealed incisor elongation after 2 weeks (n = 7). Shown is incisor elongation in representative chow- and HFD-fed mice from immediately after marking until 2 weeks later. Arrow indicates position of incisor. Scale bars = 1000 μm. (**b**) Elongation amount over 2 weeks (n = 7). Incisor elongation after 2 weeks in the HFD-fed was less than that in the chow-fed mice. (**c**) Daily elongation rate over 2 weeks in chow- and HFD-fed mice (n = 7). The daily incisor elongation rate after 2 weeks was less in the HFD-fed mice. (**d**) µ-CT sagittal section images indicating incisor pulp narrowing in 20-week-old HFD-fed mice. The incisor pulp became narrowed along with thickened dentin from the apical to incisal side. Arrow indicates pulp vertical width at mesial roots of first molar (n = 5–12). Scale bar = 1000 μm. (**e**) μCT coronal section images of incisor and sagittal section of molar (n = 5–12). Molar pulp was not narrowed in the HFD-fed mice. Scale bar = 1000 μm. (**f**) Incisor pulp, enamel, and overall vertical widths (n = 5–12). μCT image shows sites of pulp cavity vertical width, enamel vertical width, and overall vertical width measurements. Scale bar = 1000 μm. (**g**) Molar pulp, dentin, and crown width, and crown and root length (n = 5–10). Comparisons of sagittal sections of molars in μCT images of chow- and HFD-fed mice. μCT images showing sites of molar pulp width, crown width, crown length, and root length measurements. Scale bar = 1000 μm. (**b,c,f,g**) Student’s t-test, **p < 0.01, *p < 0.05. Error bars represent mean ± SD.

After 12 weeks of feeding, 20-week-old mice were examined in this study. Measurements of incisor elongation were considered to demonstrate the effects of fatty ingredients in the diet (Fig. 1a–c; Supplementary Table 1). To examine the inner structure of the incisor, we performed μCT scanning of mandibular bone specimens. The incisor pulp was narrowed in HFD-fed as compared to chow-fed mice (Fig. 1d–f), while the molar pulp was not narrowed in mice given the HFD (Fig. 1g). Additionally, time dependent representative μCT images showed an incisor pulp narrowing trend after 6 weeks of the feeding period in both HFD-fed wild-type (WT) and chow-fed *Ldlr*^*−/−*^ mice, while the incisor pulp showed a narrowing trend after 3 weeks of the feeding period in HFD-fed *Ldlr*^*−/−*^ mice (Supplementary Fig. 3). We also analyzed important hard tissue bone samples using μCT and noted that femur bone mass was decreased in HFD-fed mice (Supplementary Fig. 4a,b). These results suggested that the amount of fat consumed had effects on tooth growth and formation, as well as bone metabolism.

### Predentin in HFD-fed WT mice disappeared near central region of mandibular incisor

**Figure 2:**
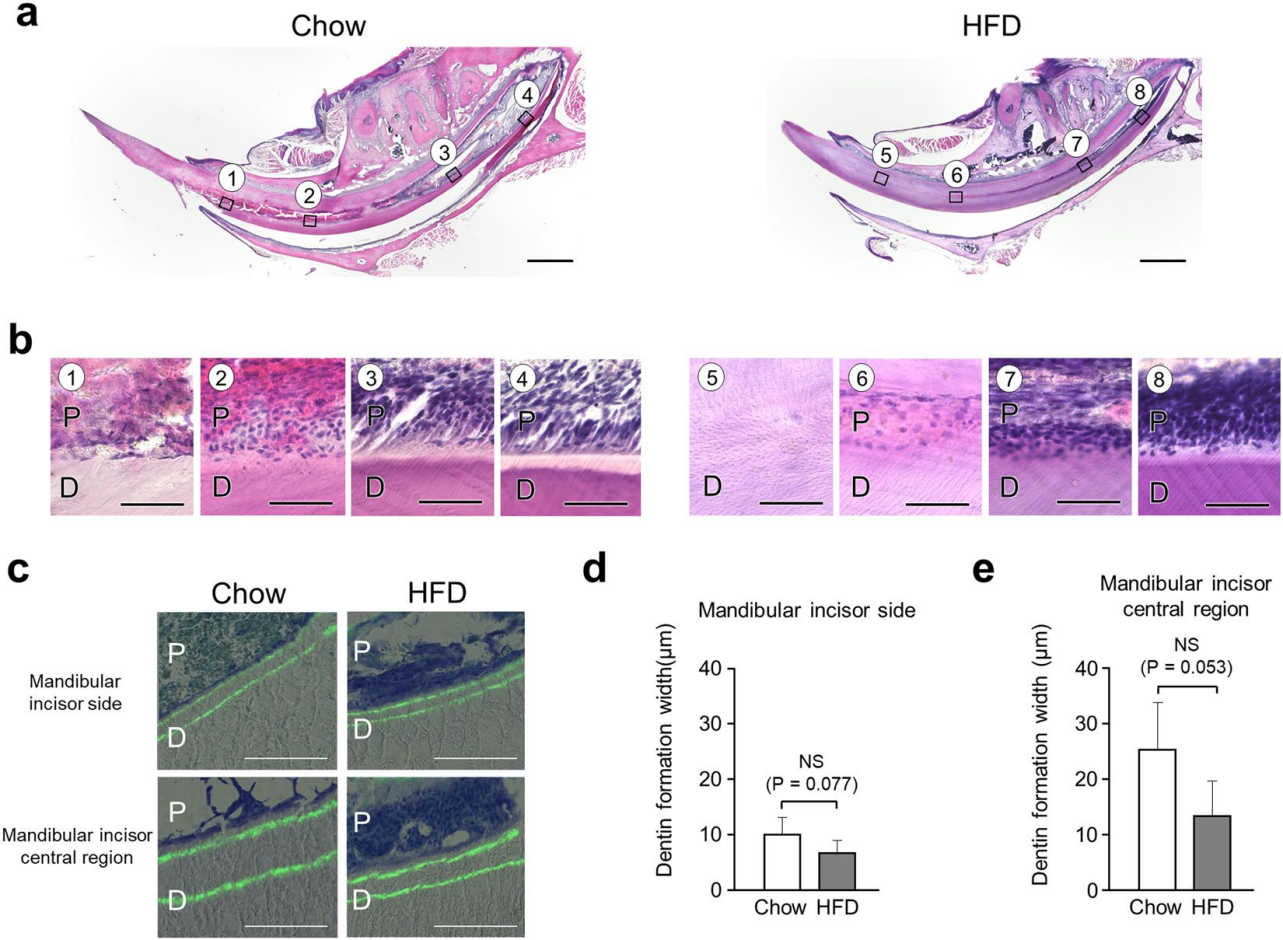
Histological analysis of WT mouse incisor dentin and pulp. (**a**) Sagittal histological section of WT mouse incisor. Scale bar = 1000 μm. (**b**) Magnified images show histological sections of pulp and odontoblasts from 20-week-old chow- and HFD-fed mice. Scale bar = 50 μm. (**c**) Calcein labeled histological section of incisal side. Scale bar = 100 μm. (**d**) Dentin formation width in mandibular incisal site (n = 5–6). (**e**) Dentin formation width in central region of mandibular incisor (n = 4–5). (**d,e**) Student’s t-test, NS; not significant. Error bars represent mean ± SD. D: dentin, P: pulp.

To perform histological examinations of narrowed incisor pulp and thickened dentin in mice given the HFD, histological sections of mandibular incisors were prepared. Sagittal section images showed that the pulp cavity of HFD-fed WT mice was significantly narrowed as compared to chow-fed WT mice (Fig. 2a), consistent with the μCT imaging findings. In addition, dentin thickness in the HFD group was significantly increased. The strong expansion shown in the images also indicated that predentin had disappeared in HFD-fed mice in the area near the central region (Fig. 2b③⑦). In addition, the morphology of odontoblasts forming the odontoblast layer was not columnar, but rather round near the central region (Fig. 2b③⑦). Also, the odontoblast layer on the apical side in HFD-fed mice showed a trend for greater density than that in chow-fed mice (Fig. 2b⑧). Dentin formation was indicated by double labelling of calcein (Fig. 2c), and the width of formed dentin showed a decreasing trend on the incisor side and in the central region of the mandibular incisor in the HFD group (Fig. 2d,e). These results suggest that the amount of fat in the diet has effects on odontoblasts during tooth formation.

### In *Ldlr*^*−/−*^ mice, incisor elongation showed no significant differences, while incisor pulp in HFD-fed mice was significantly narrowed

**Figure 3:**
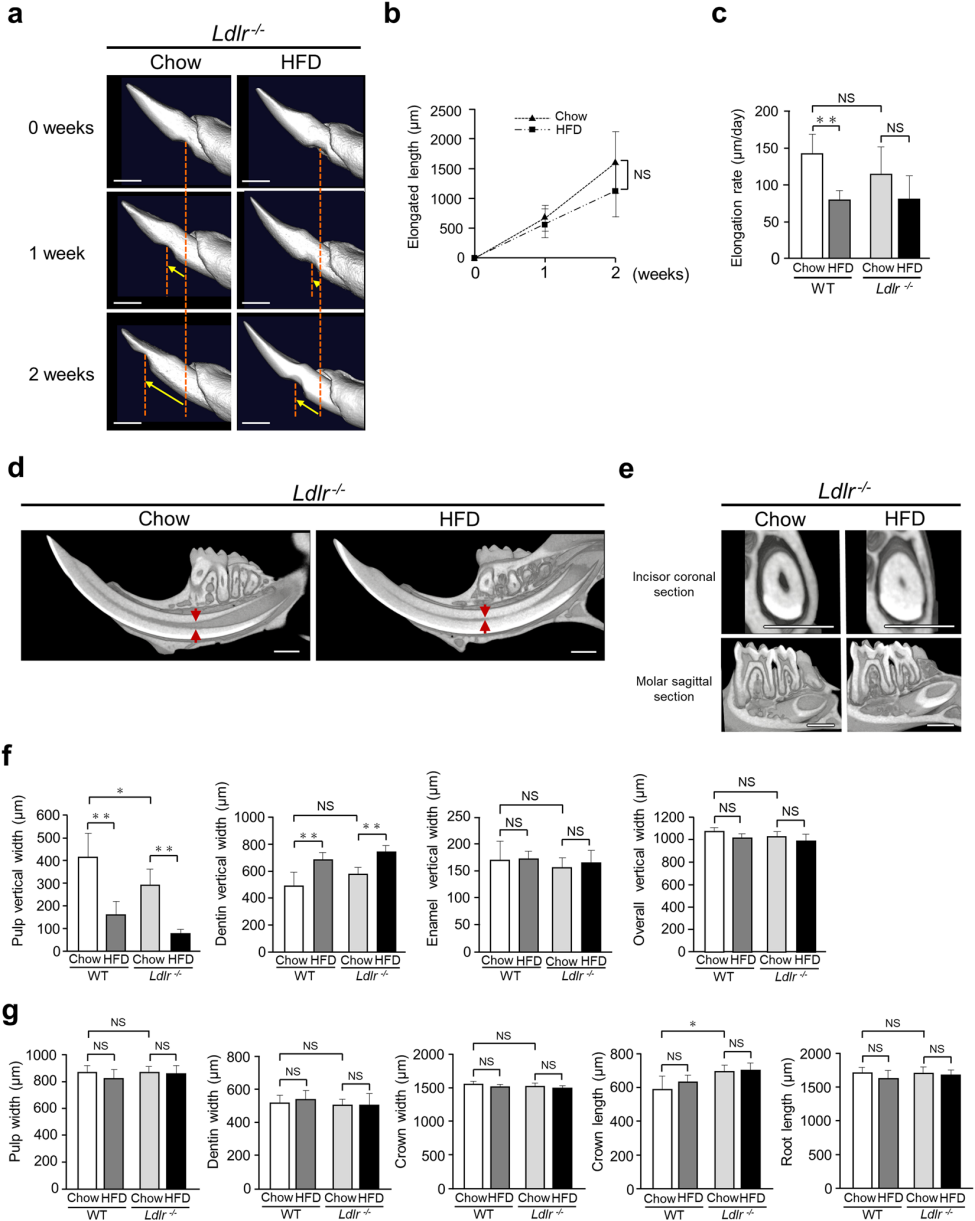
Measurements of incisor elongation in chow- and HFD-fed *Ldlr*^*−/−*^ mice. (**a**) μCT images showing incisor elongation after 2 weeks (n = 4–7). μCT time course images indicate representative incisor elongation in chow- and HFD-fed *Ldlr*^*−/−*^ mice over 2 weeks. Arrow indicates incisor marking position. Scale bars = 1000 μm. (**b**) Elongation amount after 2 weeks (n = 4–7). (**c**) Daily elongation rate for 2 weeks in WT and *Ldlr*^*−/−*^ mice. (**d**) μCT sagittal section images showing narrowed incisor pulp from apical to incisal side in 20-week-old HFD-fed as compared with chow-fed mice. Arrow indicates pulp vertical width at mesial roots of first molar (n = 7–8). Scale bar = 1000 μm. (**e**) μCT coronal images of incisor and molar sagittal sections (n = 7–8). Molar pulp narrowing was not seen in either chow- or HFD-fed mice. Scale bar = 1000 μm. (**f**) Incisor pulp, enamel, and overall vertical widths in WT and *Ldlr*^*−/−*^ mice (n = 5–12). Pulp vertical width was decreased in chow-fed *Ldlr*^*−/−*^ mice as compared to chow-fed WT mice. Incisor pulp vertical width was decreased and dentin vertical width was increased in HFD-fed *Ldlr*^*−/−*^ mice. (**g**) Molar pulp and crown width, and crown and root length in WT and *Ldlr*^*−/−*^ mice (n = 5–10). Crown length in chow-fed *Ldlr*^*−/−*^ mice was increased as compared to that in chow-fed WT mice. (f, g) One-way ANOVA with Dunnett’s test. **p < 0.01, *p < 0.05. NS, not significant. Error bars represent mean ± SD.

Body weight was increased in both chow- and HFD-fed WT mice, while that showed a gradual decrease over time in HFD-fed *Ldlr*^*−/−*^ mice. The body weight of HFD-fed WT mice was significantly increased as compared to that of HFD-fed *Ldlr*^*−/−*^ mice (Supplementary Fig. 2). To further investigate the relationships of fat in the diet with tooth growth and dentin formation, we used *Ldlr*^*−/−*^ mice. As compared to WT mice, incisor elongation amount and rate were decreased in the *Ldlr*^*−/−*^ group when given the chow diet (Fig. 3a–c), indicating that lipid metabolism by the mediated LDL receptor and other factors has effects on tooth elongation. Furthermore, incisor pulp was narrowed in HFD-fed as compared to chow-fed *Ldlr*^*−/−*^ mice (Fig. 3d–f). As for measurement results of molars in chow-fed WT and *Ldlr*^*−/−*^ mice, pulp width, dentin width, crown width, and root length showed no significant differences (Fig. 3g). When diet types were compared, femur bone mass was decreased in the *Ldlr*^*−/−*^ mice fed the HFD as compared to those given the chow diet (Supplementary Fig. 4a,b). In summary, these results suggest that the LDL receptor has some effects on tooth growth, incisor dentinogenesis, and bone metabolism.

### Predentin near central region of mandibular incisor disappeared in HFD-fed *Ldlr*^*−/−*^ mice

**Figure 4:**
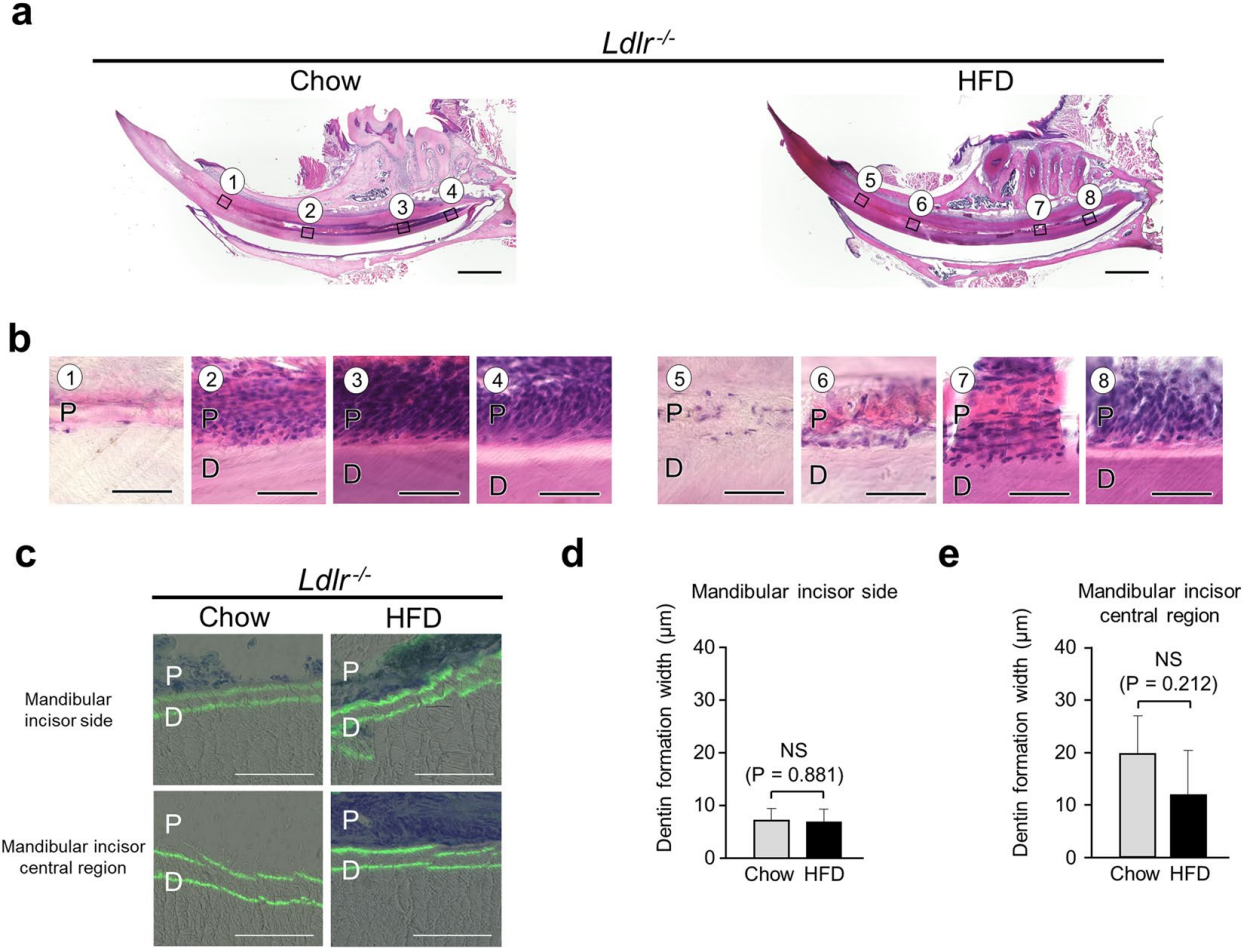
Histological analysis of incisor dentin and pulp in *Ldlr*^*−/−*^ mice. (**a**) Sagittal histological section of incisor from 20-week-old *Ldlr*^*−/−*^ mouse. Scale bar = 1000 μm. (**b**) Magnified images show histological sections of pulp and odontoblasts from chow- and HFD-fed mice. Scale bar = 50 μm. **(c**) Calcein labeled histological section of incisor. Scale bar = 100 μm. (**d**) Dentin formation width in mandibular incisor in WT and *Ldlr*^*−/−*^ mice (n = 3–6). (**e**) Dentin formation width in central region of mandibular incisor in WT and *Ldlr*^*−/−*^ mice (n = 3–5). (**d,e**) One-way ANOVA with Dunnett’s method. NS, not significant. Error bars represent mean ± SD. D: dentin, P: pulp.

Examinations of sagittal histological sections showed narrowed incisor pulp with thickened dentin in both HFD- and chow-fed *Ldlr*^*−/−*^ mice (Fig. 4a). On the other hand, strong expansion seen in imaging findings and predentin near the central region of the mandibular incisor had disappeared in the HFD-fed *Ldlr*^*−/−*^ mice (Fig. 4b⑦). In addition, the morphology of odontoblasts near the central region in chow-fed *Ldlr*^*−/−*^ mice indicated a more circular shape as compared to chow-fed WT mice (Fig. 2b③, Fig. 4b③). Also, the odontoblast layer on the apical side in both chow- and HFD-fed *Ldlr*^*−/−*^ mice demonstrated a trend for greater density as compared to the WT mice given chow (Fig. 2b④, Fig. 4b④⑧). Dentin formation widths on the mandibular incisor side and in the central region of the mandibular incisor were nearly the same in the chow- and HFD-fed *Ldlr*^*−/−*^ mice (Fig. 4c–e). These results suggested that tooth constituent cells in the *Ldlr*^*−/−*^ mice were also affected by fat in the diet and the LDL receptor.

### Ameloblasts near central region of mandibular incisor in HFD-fed WT mice, and chow- and HFD-fed *Ldlr*^*−/−*^ mice promoted differentiation stage

**Figure 5:**
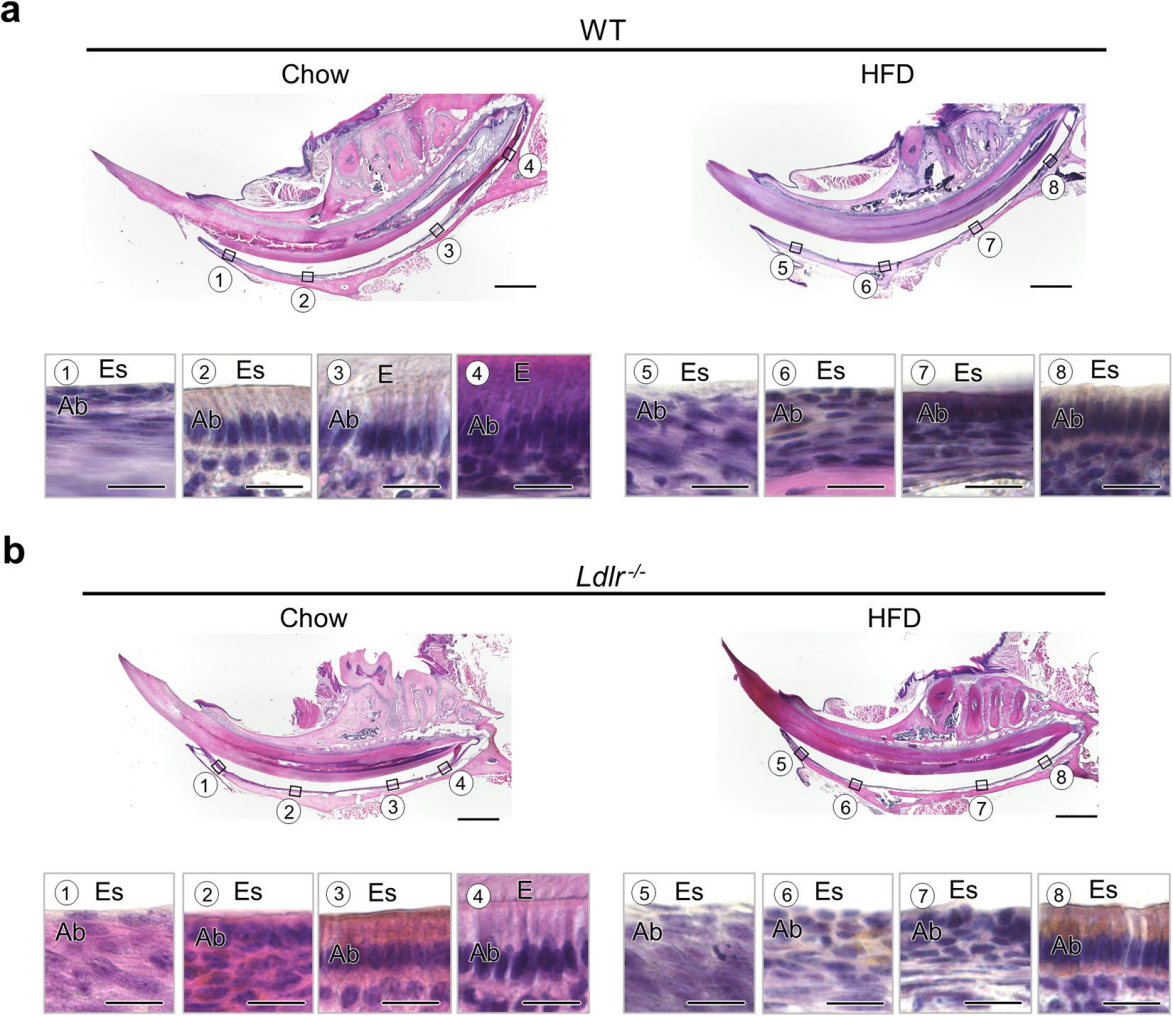
Histological analysis of incisor ameloblasts in WT and *Ldlr*^*−/−*^ mice. (**a**) Sagittal histological section of incisor from 20-week-old WT mouse (upper panel). Scale bar = 1000 μm. Magnified image of ameloblast (lower panel). Scale bar = 30 μm. (**b**) Sagittal histological section of incisor from 20-week-old *Ldlr*^*−/−*^ mouse (upper panel). Scale bar = 1000 μm. Magnified image of ameloblast (lower panel). Scale bar = 30 μm. E: enamel, Es: enamel space, Ab: ameloblast.

Images showing strong expansion indicated a cuboidal ameloblast morphology near the central region in the HFD-fed WT, and chow- and HFD-fed *Ldlr*^*−/−*^ mice, as compared to the chow-fed WT mice (Fig. 5a②⑥, 5b②⑥). These results suggested that the ameloblast differentiation process was promoted in the HFD-fed WT, and chow- and HFD-fed *Ldlr*^*−/−*^ mice.

### *Ldlr*^*−/−*^ mice given HFD developed severe dyslipidemia

**Figure 6:**
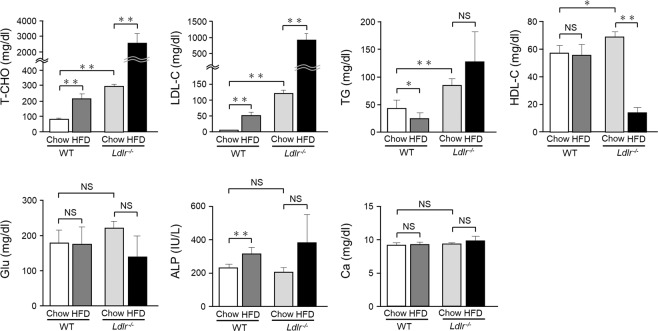
WT and *Ldlr*^*−/−*^ mouse blood analysis. Blood was obtained from 20-week-old WT and *Ldlr*^*−/−*^ mice (n = 4–12), and examined. T-CHO: total cholesterol, LDL-C: low density-cholesterol, HDL-C: high density-cholesterol, TG: triglyceride, Glu: glucose, ALP: alkaline phosphatase, Ca: calcium. One-way ANOVA with Dunnett’s test. **p < 0.01, *p < 0.05. NS, not significant. Error bars represent mean ± SD.

In the HFD-fed WT mice, the levels of total-cholesterol (T-CHO), low density lipoprotein-cholesterol (LDL-C), and alkaline phosphatase (ALP) were increased by 2.6-, 10-, and 1.4-fold, respectively, as compared to WT mice given the chow diet, while the level of triglycerides (TG) was decreased in WT mice given the HFD (Fig. 6). On the other hand, the levels of high density lipoprotein-cholesterol (HDL-C), glucose (Glu), and calcium (Ca) in WT mice showed no significant differences between the diet groups (Fig. 6). In contrast, in the HFD-fed *Ldlr*^*−/−*^ mice, the levels of T-CHO and LDL-C were significantly increased, while the level of HDL-C was decreased as compared to those given chow (Fig. 6), whereas the levels of TG, Glu, ALP, and Ca showed no significant differences between *Ldlr*^*−/−*^ mice with the different diets (Fig. 6). Comparisons of chow-fed WT and *Ldlr*^*−/−*^ mice showed that the levels of T-CHO (3.7-fold), LDL-C (24-fold), and TG (2-fold) were increased in the latter group (Fig. 6). These results indicated that increased fat in the diet induced an increase in T-CHO and LDL-C levels in both the WT and *Ldlr*^*−/−*^ mice (Fig. 6), which resulted in narrowed pulp and thickened dentin in incisors.

## Discussion

The present results showed that a diet high in fat has effects on tooth formation and growth, as well as bone metabolism, suggesting involvement of lipid components, especially cholesterol and LDL. In mice, incisors are different than molars, as they continue to grow and elongate throughout life. A previous study showed that measurement of incisor eruption rate is an appropriate method for evaluation of tooth physiological growth^32^, thus we mainly examined incisors in the present study. Our findings showed that the amount of elongation was decreased in WT mice fed the HFD (Fig. 1), while a decreasing trend was found in the HFD-fed *Ldlr*^*−/−*^ mice (Fig. 3), suggesting that a lipid metabolism disorder can delay tooth growth. In a previous study, a decrease in blood flow was found to induce a decreased incisor eruption rate in mice^33^, while another reported that *Ldlr*^*−/−*^ mice fed an HFD showed plaque adherence in the aorta^34^. Together, these results suggest that peripheral blood vessels such as those in pulp also have decreased blood flow, which impairs elongation of the incisor. To better understand the effects of lipid metabolism on tooth growth, a more detailed examination of molars will be necessary.

In the present experiments, the amount of dentin in the incisors of both WT and *Ldlr*^*−/−*^ mice given the HFD was notably increased (Figs. 1 and 3), while a slight increase was seen in the chow-fed *Ldlr*^*−/−*^ mice (Fig. 3). In addition, time-dependent μCT images showed that incisor pulp gradually narrowed in dyslipidemia model mice (Supplementary Fig. 3). In the chow-fed *Ldlr*^*−/−*^ mice, the levels of T-CHO and LDL-C were increased as compared to chow-fed WT mice (Fig. 6), suggesting that increased cholesterol in blood induced narrowing of pulp with thickened dentin.

A physiological increase in dentin is known to normally occur throughout life, though some reports have noted increased dentin due to local or systemic pathological factors. Regarding local factors, attrition, abrasion, foreign materials, and caries, as well as other factors have been reported to cause an increase in dentin in both incisors and molars^35,36^. As for systemic factors, some patients who continue to receive steroid administration for long periods after undergoing transplantation for renal disease have shown a significantly narrowed dental pulp cavity in both incisors and molar teeth^37,38^, while those patients often develop dyslipidemia secondarily as well^39,40^. A previous study reported that dyslipidemia model mice had a narrowed incisor dental pulp cavity^41^. Others that investigated the relationship between leptin and the LDL receptor also showed that HFD consumption induced increased plasma leptin levels in both WT and *Ldlr*^*−/−*^ mice^42,43^, while it is known that leptin promotes odontoblastic differentiation^44,45^. Together, these findings indicate that an increased plasma leptin level induces narrowed pulp with thickened dentin in WT mice. However, chow-fed *Ldlr*^*−/−*^ mice in a previous study showed no increased level of leptin in plasma^43^. Furthermore, deletion of the sphingomyelin phosphodiesterase 3 (*Smpd3*) gene was found to impair dentinogenesis and osteogenesis in alveolar bones in mice^20^, thus *Smpd3* is considered to be an important factor for dentinogenesis. We consider that the mechanism underlying the present results may involve additional factors other than leptin.

It is thus suggested that increased lipid biomarkers, including cholesterol, as well as hormones related to lipid metabolism, including leptin, in blood induce tooth pulp narrowing. Narrowed pulp cavities have been found in both incisors and molars in humans, whereas only incisors were affected in the present mice, likely because of histological and anatomical differences between humans and rodents. As noted above, a rodent incisor shows growth throughout life, whereas dentin in humans increases because of physiological reasons or when stimulated. Also, active mesenchymal stem cells providing new tooth constituent cells to maintain growth have been found in mouse incisors^46,47^.

It is also considered that reactive cells are more susceptible to an increased fat component in the diet as compared to quiescent cells. In the present study, calcein labeling showed that dentin formation on the incisal side and in the central region was decreased in teeth considered to be in an active formation state (Figs. 2d,e and 4d,e), suggesting that dentin formation ability was declining at 12 weeks after beginning the special diet. Histological sections also showed that odontoblast density in dentin was increased in the HFD-fed WT and *Ldlr*^*−/−*^ mice, as well as chow-fed *Ldlr*^*−/−*^ mice (Figs. 2b and 4b), indicating that odontoblast differentiation and/or function was increased in those mice. Therefore, it is suggested that the mechanism of dentin formation promotion differs between WT and *Ldlr*^*−/−*^ mice. However, the pathway involving the LDL receptor might not be exclusive and different pathways may also be involved.

Histological findings showed that pre-dentin in the HFD-fed WT and *Ldlr*^*−/−*^ mice disappeared (Figs. 2 and 4), and the odontoblast morphology was circular within that region, while pre-dentin was unclear in *Ldlr*^*−/−*^ mice given chow. In addition, ameloblast morphology was cuboidal near the central region in the HFD fed WT, and chow- and HFD- fed *Ldlr*^*−/−*^ mice (Fig. 5a②⑥, 5b②⑥). These results suggest that increased fat in the diet induces morphological changes in odontoblasts and ameloblasts, as well as dentin formation ability and ameloblast differentiation, while a deficiency of the LDL receptor has effects on dentin formation and the process of ameloblast differentiation. μCT imaging of femurs showed that bone mass was decreased in WT mice fed the HFD as compared to chow, while the *Ldlr*^*−/−*^ mice showed no significant difference regardless of diet (Supplementary Fig. 4). However, the bone volume/tissue volume (BV/TV) ratio in *Ldlr*^*−/−*^ mice fed chow had a decreasing trend as compared to the chow-fed WT mice, while that ratio was equally decreased in the HFD-fed *Ldlr*^*−/−*^ and WT mice. A previous study demonstrated that LDL is associated with hard tissue metabolism, especially oxidized LDL, which promotes bone loss by facilitating adipogenic differentiation more than osteogenic differentiation in bone marrow stromal cells^48^. Our results indicate that increased cholesterol in blood induces a decrease in bone mass, while histological examinations of femur sections revealed fatty bone marrow (data not shown), thus suggesting a mechanism of decreased bone formation in mice with increased cholesterol. Achilles tendon thickness, calcification, and FH are also known to have associations^49,50^. In addition, ultrastructural analysis of odontoblasts, ameloblasts, dental pulp and extracellular matrixes in the teeth is necessary to clarify the mechanisms for the effects of lipid metabolism on tooth development. In the present mice, teeth and bones showed changes, indicating that tissue subjected to stress has increased collagen.

In summary, the present results provided clarification regarding the role of the LDL receptor in dentin formation and bone metabolism. In addition, effects of cholesterol and the LDL receptor on dentin formation and bone metabolism were suggested.

## Methods

### Mice and feeding

WT mice with a C57BL/6J genetic background were purchased from Sankyo Labo Service Corporation, Inc. Tokyo, Japan. The *Ldlr*^*−/−*^ (B6. *Ldlr*^*tm1Her*^/J) mouse strain was purchased from The Jackson Laboratory (stock no. 2207). At 8 weeks old, all mice were separated into those fed regular chow (CRF-1; 24.8% protein, 13.7% fat, 61.5% carbohydrate; Oriental Yeast Corp., Tokyo, Japan) or F2HFD1 (HFD; 22% protein, 36% fat, 42% carbohydrate; Oriental Yeast Corp., Tokyo, Japan). Details regarding the compositions of the diets are shown in Supplementary Table 1. After being fed chow or the HFD for 12 weeks, blood, skull bone, and mandibular bone samples were collected following administration of a mixed anesthetic (midazolam, medetomin, vetorphale). The mice were labeled with calcein via a subcutaneous injection at 5 days and again 1 day before euthanasia. The experimental design is shown in Supplementary Fig. 1. All animal experiments were approved by the Showa University Animal Care and Use Committee (approval number: 19009), and conducted according to the ethical guidelines of that institution.

### Incisor elongation rate

At the age of 18 weeks, the mandibular right incisor cervix surface in each mouse was defaced using a power pack (Minimo ONE SEREIES ver. 2; MINITOR CO., Ltd. Tokyo, Japan), standard rotary (M212H; MINITOR CO., Ltd. Tokyo, Japan), and 1-mm round bur, with the mice given the same mixed anesthetic noted above. Tooth elongation process X-ray photography was performed with an *in vivo* μCT system (R_mCT2; Rigaku Co., Ltd., Tokyo, Japan) at 1 and 2 weeks after defacement^51^. Incisor elongation rate was determined by measuring from the alveolar bone crest to middle of the round lesion using the Image J software package, version 1.52a (National Institutes of Health, Maryland, United States).

### Micro-computed tomography (μCT) analysis

Mandibular bone and femur specimens obtained from the mice were scanned using a ScanXmate-L090H (Comscantecno, Co., Ltd., Yokohama, Japan), as previously described^52^. Three-dimensional distal images were reconstructed with a TRI/3D-Bon-FCS system (RATOC System Engineering Co., Ltd., Tokyo, Japan). Following reconstruction, the coronal plane at the mesial root of the first molar was selected to determine enamel, dentin, and tooth full-width thicknesses, and pulp cavity diameter, with the values calculated using Image J 1.52a.

### Mandibular incisor histological sections

Mandibular bone specimens obtained from the mice were fixed in a 4% paraformaldehyde phosphate buffer solution (FUJIFILM Wako Pure Chemical Industries, Ltd., Osaka, Japan) or 10% formalin neutral buffer solution (FUJIFILM Wako Pure Chemical Industries, Ltd., Osaka, Japan), then demineralized for 2 weeks in decalcification solution (OSTEOSOFT; Merck KGaA, Corp., Damstadt, Germany). Thereafter, they were embedded in paraffin wax and cut into sagittal sections, and subjected to hematoxylin and eosin staining. All sections were analyzed using an all-in-one fluorescence microscope (BZ-X710; KEYENCE, Corp., Osaka, Japan).

### Serum examinations

After completion of the 12-week special feeding period, all mice were euthanized and blood samples were collected from the heart. Serum levels of total cholesterol (T-CHO), low density lipoprotein cholesterol (LDL-C), high density lipoprotein cholesterol (HDL-C), triglyceride (TG), calcium (Ca), inorganic phosphorus (IP), glucose (Glu), and alkaline phosphatase (ALP) were determined using routine laboratory methods (Oriental Yeast Corp., Tokyo, Japan).

### Statistical analysis

Statistical analyses were performed with the IBM PASW statistical software package, version 18.0 (IBM, Chicago IL, USA). Values are expressed as the mean ± SD (standard deviation). All analyses were performed using one-way ANOVA or Student’s *t*-test, with *p* values less than 0.05 considered to indicate statistical significance.

## Supplementary information

Supplementary Information.
