# Supplementary material for: Effects of lipid metabolism on mouse incisor dentinogenesis
Source: Sci Rep. 2020 Mar 20;10:5102. doi: 10.1038/s41598-020-61978-0 (PMC7083963; doi:10.1038/s41598-020-61978-0)
Supplement: Supplementary file 1 — Supplementary Information. [file 41598_2020_61978_MOESM1_ESM.pdf]

Effects of Lipid Metabolism on Mouse Incisor Dentinogenesis

Yutaro Kurotaki[1, 2, 3], Nobuhiro Sakai[2, 3]*, Takuro Miyazaki[4], Masahiro Hosonuma[2, 3, 5], Yurie Sato[2, 3, 6], Akiko Karakawa[2, 3], Masahiro Chatani[2, 3], Mie Myers[1], Tetsuo Suzawa[7], Takako Negishi-Koga[2, 3, 8], Ryutaro Kamijo[7], Akira Miyazaki[4], Yasubumi Maruoka[1], and Masamichi Takami[2, 3]*

[1] Division of Community-Based Comprehensive Dentistry, Department of Special Needs Dentistry, School of Dentistry, Showa University; 2–1–1 Kitasenzoku, Ota, Tokyo 145–8515, Japan

[2] Department of Pharmacology, School of Dentistry, Showa University; 1–5–8 Hatanodai, Shinagawa, Tokyo, 142–8555, Japan

[3] Pharmacological Research Center, Showa University; 1–5–8 Hatanodai, Shinagawa, Tokyo, 142–8555, Japan

[4] Department of Biochemistry, School of Medicine, Showa University; 1–5–8 Hatanodai, Shinagawa, Tokyo, 142–8555, Japan

[5] Division of Rheumatology, Department of Medicine, Showa University; 1–5–8 Hatanodai, Shinagawa, Tokyo, 142–8555, Japan

[6] Division of Dentistry for Persons with Disabilities, School of Dentistry, Showa University; 2–1–1 Kitasenzoku, Ota, Tokyo 145–8515, Japan

[7] Department of Biochemistry, School of Dentistry, Showa University; 1–5–8 Hatanodai, Shinagawa, Tokyo, 142–8555, Japan

[8] Division of Mucosal Barriology, International Research and Development Center for Mucosal vaccines, The Institute of Medical Science, The Institute of Medical Science The University of Tokyo; 4-6-1 Shirokanedai, Minato, Tokyo, 108-8639, Japan.

*Corresponding authors:

Nobuhiro Sakai

Department of Pharmacology, School of Dentistry, Showa University; 1–5–8 Hatanodai, Shinagawa, Tokyo, 142–8555, Japan

Email: nobsakai@dent.showa-u.ac.jp

Masamichi Takami

Department of Pharmacology, School of Dentistry, Showa University; 1–5–8 Hatanodai, Shinagawa, Tokyo, 142–8555, Japan

Email: takami@dent.showa-u.ac.jp

Keywords: incisor elongation, odontoblast, high-fat diet, cholesterol, LDL receptor

Supplementary Table 1. Diet composition.

	Chow	HFD
1. Nutrition contents (within 100 g)		
Total calories (Kcal)	357	414
Fat (g)	5.4	16.5
Protein (g)	21.9	22.6
Carbohydrates (g)	55.3	45.1
2. Estimated calorie ratio		
Fat (%)	13.7	36
Protein (%)	24.8	22
Carbohydrates (%)	61.5	42

Chow = CRF-1, HFD = F2HFD1. Higher calorie and fat levels in HFD.

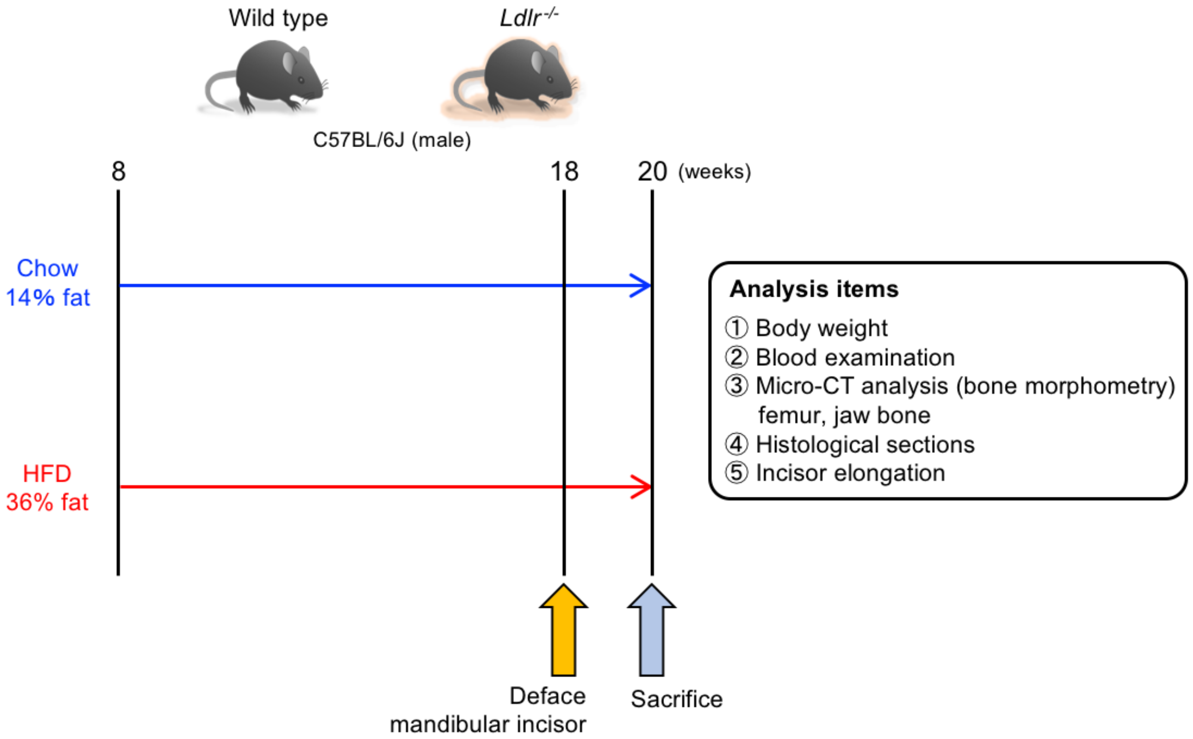

Supplementary Figure 1. Schematic diagram of experimental plan.

WT mice and *Ldlr* $^{-/-}$ mice were fed chow or the HFD from 8 to 20 weeks of age. At 18 weeks, the mandibular incisor was defaced, then samples were collected 2 weeks later.

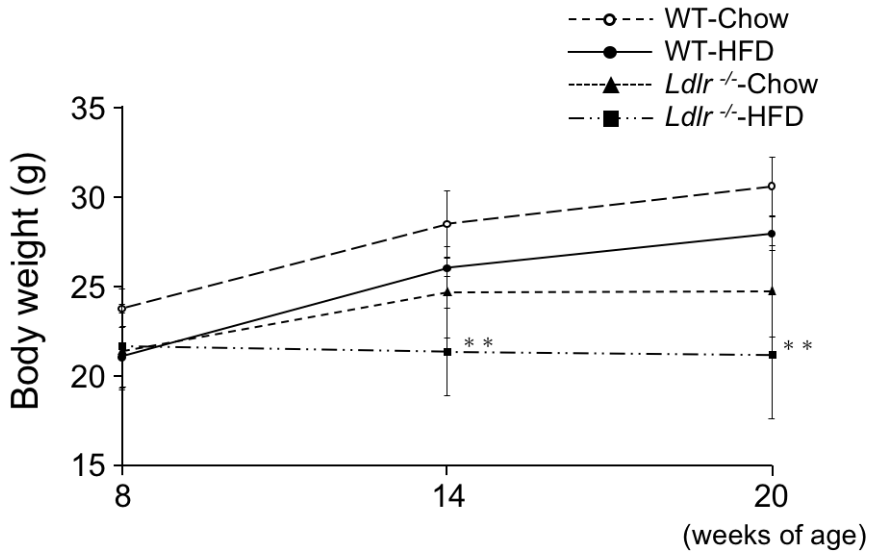

Supplementary Figure 2. Time course examination of body weight.

Graph shows time course changes in body weights of WT and *Ldlr*$^{-/-}$ mice every 6 weeks (n = 4-9). Body weight was increased in both chow- and HFD-fed WT mice. In HFD-fed *Ldlr*$^{-/-}$ mice, body weight showed a gradual decrease over time. The body weight of HFD-fed WT mice was significantly increased as compared to HFD-fed *Ldlr*$^{-/-}$ mice. One-way ANOVA with Dunnett's test. **p <0.01 vs. HFD-fed WT. Error bars represent mean ± SD.

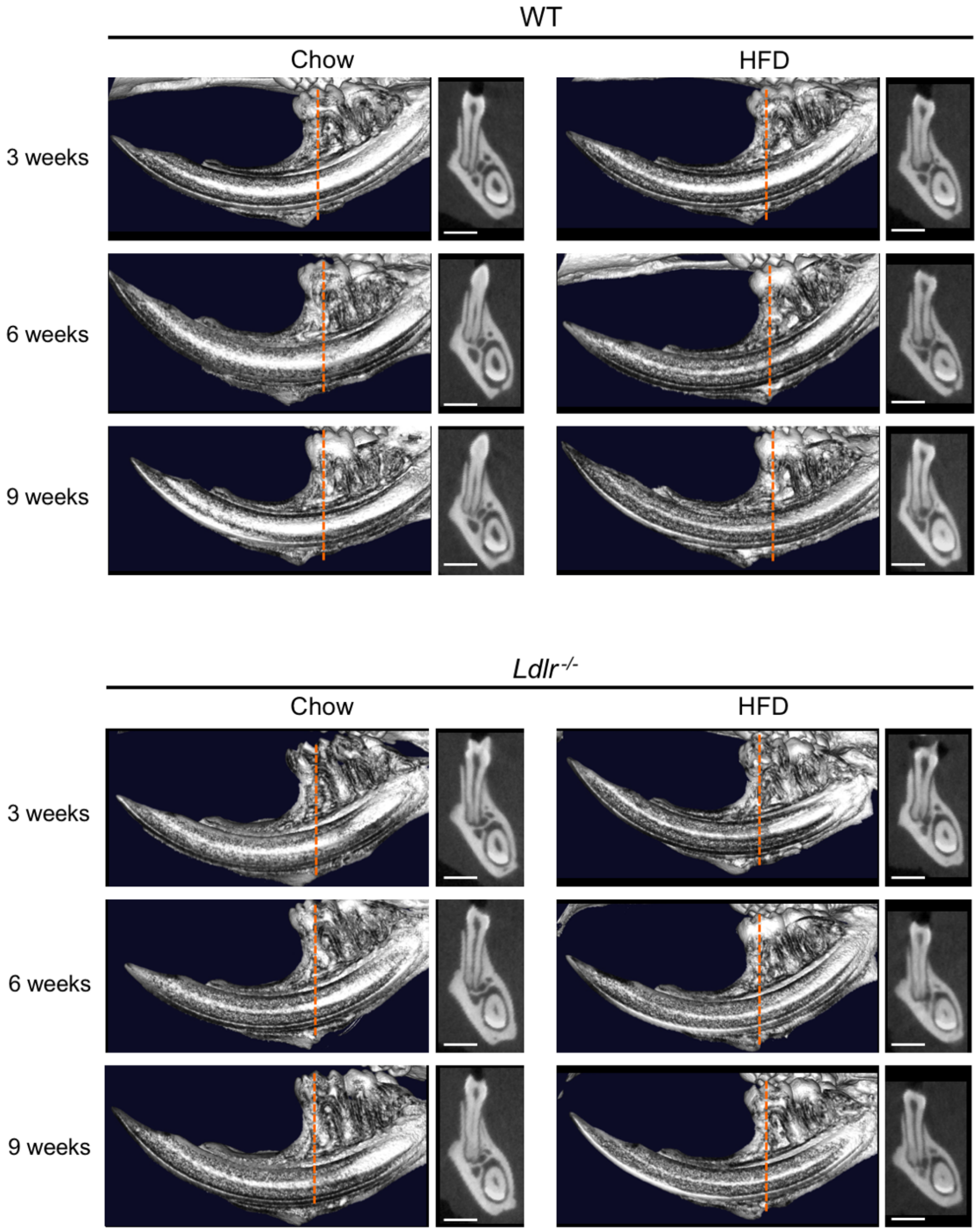

Supplementary Figure 3. Representative μCT images of mandibular incisor changes over time. Upper shows sagittal section, lower shows coronal section. μCT images show incisor pulp state. Scale bar = 1000 μm.

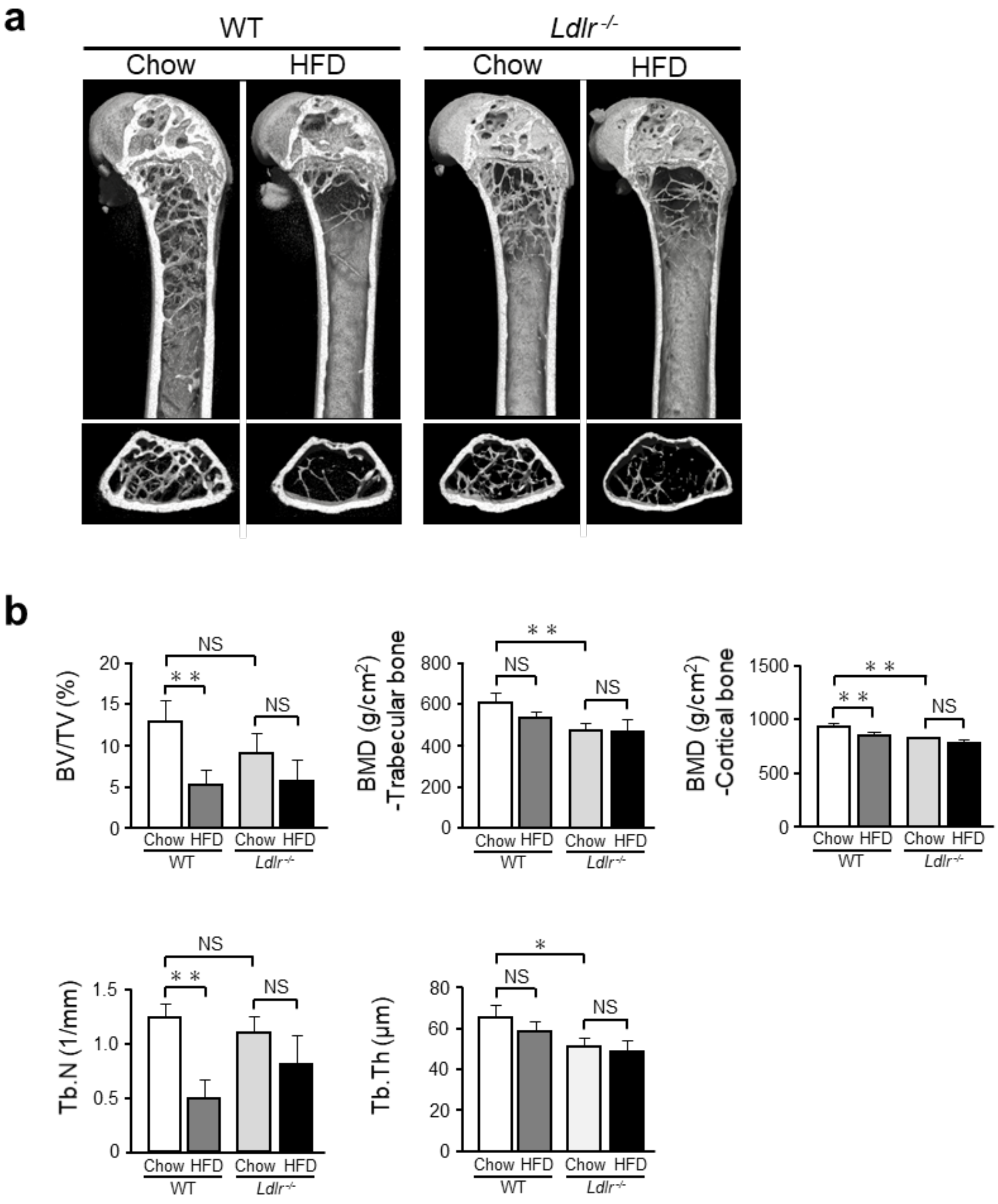

Supplementary Figure 4. Decreased bone mass in HFD-fed WT and *Ldlr*$^{-/-}$ mice.

(a) μCT images of femur. Upper shows sagittal section, lower shows transverse section (n = 4-6). Three-dimensional imaging of femurs revealed decreased bone mass in HFD-fed as compared to chow-fed mice. μCT imaging of femurs also showed decreased trabecular bone in both chow- and HFD-fed *Ldlr*$^{-/-}$ mice as compared to chow-fed WT mice. (b) Bone morphometry analysis (n = 4-6). BV/TV: bone volume to tissue volume ratio, BMD-trabecular bone: bone mineral density-trabecular bone, BMD-C: bone mineral density, Tb. N: trabecular number, Tb. Th: trabecular thickness. Bone morphometry results showed decreased BV/TV, BMD-C, and Tb. N in HFD-fed mice, while there were no significant differences for BMD-T and Tb. Th. Bone morphometry results showed that BMD-T, BMD-C, and Tb. Th were decreased in chow-fed *Ldlr*$^{-/-}$ mice as compared to chow-fed WT mice, while there were no significant differences for BV/TV and Tb. N. (b) One-way ANOVA with Dunnett's test. ***p* <0.01, **p* <0.05. NS, not significant. Error bars represent mean ± SD.
